# Association between lymphocyte count in bronchoalveolar lavage fluid and mortality

**DOI:** 10.1186/s40560-021-00568-2

**Published:** 2021-09-15

**Authors:** Yifu Si, Juqin Shao, Caibao Hu

**Affiliations:** 1 Department of Internal Medicine, Pinghu First People’s Hospital, 500#, Sangang-Road, Pinghu, Zhejiang China; 2 Department of Intensive Care, Zhejiang Hospital, 1220#, Gudun-RoadZhejiang, Hangzhou, 310030 China

**Keywords:** Acute respiratory failure, Bronchoalveolar lavage fluid, Interstitial lung disease, Lymphocyte

## Abstract

This is a comment on the paper by Dr. Hirasawa et al. on the predictive value of lymphocyte counts in bronchoalveolar lavage fluid in patients with acute respiratory failure.

## To the Editor,

We read with great interest Dr. Hirasawa et al.’s study [1], which investigated the predictive value of lymphocyte counts in bronchoalveolar lavage fluid (BALF) in patients with acute respiratory failure (ARF). This study is well designed. However, several issues should be noted when interpreted these findings.

First, ARF is a heterogeneous syndrome with various etiologies. The predictive ability of lymphocytes in BALF may be different in different diseases. For instance, in the current study, almost half of the included patients (38/78) had a diagnosis of interstitial lung disease (ILD). According to the American Thoracic Society clinical practice guideline [2], lymphocytic cellular pattern (lymphocyte count > 15% in BALF) is common in ILD. Whether the cutoff value of lymphocytes is different from other diseases remains unclear. In addition, studies are reporting that the lymphocyte in BALF did not show significant predictive value in different diseases. In a study of corona virus disease 2019 [3], Takei et al. reported that patients with high or low BALF lymphocyte (> 25 vs. < 25%) had comparable disease severity either on intensive care unit admission or on the day of BALF extraction. In a study of non-cystic fibrosis bronchiectasis [4], the lymphocyte count in BALF was comparable between the infection and non-infection groups. The stability of these findings [3, 4] may be uncertain due to the relatively small sample size. However, whether the current findings applied to ARF patients caused by different diseases also need to be investigated in future studies.

In addition, in the current study, ILD is associated with decreased mortality. This is a little unexpected. In another multicenter observational study including four different cohorts [5] (*n* = 2633, 5320, 2068, and 1670, respectively), interstitial lung abnormalities were consistently associated with a higher risk of all-cause mortality. There is a possibility that this difference was caused by the small sample size. However, if this is the case, the current finding would be biased. For instance, if the presence of ILD was associated with increased mortality in a real world, there should be more ILD patients in the non-survivor group in the current study, which may be missed due to sampling error. In this case, the association between lymphocyte and mortality might be biased.

## Data Availability

Not applicable.

## Abbreviations

ARF: Acute respiratory failure
BALF: Bronchoalveolar lavage fluid
ILD: Interstitial lung disease
